# ﻿*Impatiensmeishanensis* (Balsaminaceae), a new species from Sichuan, China

**DOI:** 10.3897/phytokeys.260.150685

**Published:** 2025-07-28

**Authors:** Yanru Zhang, Xinyu Chen, Tianmeng Qu, Xinyi Zheng, Bo Li, Jieli Yue, Xiaoqin Liu, Min Pan, Ke Huang, Zhixi Fu

**Affiliations:** 1 Key Laboratory of Land Resources Evaluation and Monitoring in Southwest (Sichuan Normal University), Ministry of Education, Chengdu 610101, China; 2 College of Life Sciences, Sichuan Normal University, Chengdu 610101, China; 3 Sichuan Ecological Environment Monitoring Station, Chengdu 610091, China; 4 Meishan Eco-environmental Monitoring Center Station of Sichuan Province, Meishan, 620010, China; 5 Sustainable Development Research Center of Resources and Environment of Western Sichuan, Sichuan Normal University, Chengdu 610101, China

**Keywords:** *Impatiens*, new species, morphology, phylogeny

## Abstract

*Impatiensmeishanensis* K.Huang & Z.X.Fu, **sp. nov.**, a new species of Balsaminaceae from Meishan City, Sichuan Province, China, is described and illustrated. The complete chloroplast (cp) genome of *I.meishanensis* is 152,104 bp in length. Based on phylogenetic analysis of the complete cp genome, *I.meishanensis* represents a distinct species closely related to *Impatiensfaberi* Hook.f. The new species can be distinguished from the latter by its unique morphological characteristics: lower sepal gable-boat-shaped (vs. funnel-shaped), spur straight (vs. curved), flower large, ca. 4 cm long (vs. ca. 3 cm long), and seeds ellipsoid with reticulate seed coat ornamentation (vs. oblong, smooth). In addition, a distribution map, a detailed morphological comparison with related species, and an assessment of the conservation status of this newly identified species are provided.

## ﻿Introduction

The genus *Impatiens* (Balsaminaceae) is notable for its vibrant colors and high ornamental value. Additionally, it holds significant importance in the medical field (Chua 2016; Li et al. 2022). Globally, there are over 1,000 species of *Impatiens* (Yu et al. 2010). These are primarily distributed across the tropical and subtropical mountainous regions of the Old World, with five main diversity centers: Tropical Africa, Madagascar, the Eastern Himalayan region, southern India and Sri Lanka, and Southeast Asia in a broad sense (Myanmar, Thailand, southwestern China, the Central and South China Peninsulas, and the Malay Archipelago) (Song et al. 2003; Yuan et al. 2004; Mabberley 2017). In China, more than 352 species of wild *Impatiens* have been identified, with the major distribution in Yunnan, Sichuan, Xizang, and Guizhou (Yuan et al. 2022a). The semi-succulent stems, fleshy leaves, and extremely fragile flowers of the genus make it difficult to classify them morphologically (Narayanan et al. 2013; Utami 2013; Luo et al. 2014).

In recent years, several new *Impatiens* species from China have been reported. It includes *Impatiensbijieensis* X.X. Bai & L.Y. Ren (Ren et al. 2022), *Impatiensguiqingensis* S.X.Yu (Guo et al. 2016), *Impatiensliupanshuiensis* X.X. Bai & T.H. Yuan (Yuan et al. 2022b), *Impatiensplicatisepala* C.Y. Zou, Yan Liu & S.X. Yu (Zou CY et al. 2020), *Impatienswawuensis* B. Ding & S.X. Yu (Ding et al. 2016), and *Impatienswutaishanensis* R.L. Liao & Lei Cai (Liao et al. 2021) *piufanensis*. In addition, in this study, the complete cp genome was sequenced and used to identify the phylogenetic relationship of new species and their relatives. Many new species have been jointly supported by genetic and morphological data, including *Impatiensbeipanjiangensis* Jian Xu & H.F. Hu (Hu et al. 2024), *Impatienschenmoui* Zheng W. Wang, Xiao C. Li & Qi Wang bis (Wang et al. 2022), *Impatiensmacrantha* S.X. Yu & Ying Qin (Qin et al. 2020), *Impatiensyingjingensis* Xin Q. Song, B.N. Song & Biao Yang (Song et al. 2024), and *Impatiensyunlingensis* S.X. Yu, Chang Y. Xia & Jiang H. Yu (Yu et al. 2022). These findings not only enriched the species diversity of the *Impatiens* but also provided ideas for further studies on its phylogeny and geographical distribution.

In early September 2024, during field monitoring in the Wawu Mountain scenic area of Meishan City, Sichuan Province, a special *Impatiens* species was collected and investigated. It was small in popularity and similar to the *Impatiensdistracta* Hook.f. The new species exhibited the peculiarity that the flower was larger, the lower sepal was formed closer to the dorsal petal, and the spur was straight. However, it does not match any of the already described species after extensive morphological comparison and phylogenetic analysis. It found that the plant might be a new species in the genus *Impatiens*. The morphology and molecular data were collected and employed to identify its relatives.

Based on morphological and molecular evidence, Yu et al. (2016) proposed a new taxonomic system of *Impatiens*, featuring two subgenera (*Clavicarpa* and *Impatiens*). The subg. Impatiens was further subdivided into seven sections (*Fasciculatae*, *Impatiens*, *Racemosae*, *Scorpioidae*, *Semeiocardium*, *Tuberosae*, and *Uniflorae*). The sect. Impatiens are characterized by two-ﬂowered inﬂorescences, linear capsule, ellipsoid seeds, and seed coats with reticulate ornamentation (Yu et al. 2016). This new species belongs to the sect. Impatiens of the subg. Impatiens.

In this study, it provided a detailed description and taxonomic position of this new species by morphological characters and molecular phylogenetic analyses. The distribution map, habitat photographs, detailed morphological images, and deep comparisons with related species were also presented.

## ﻿Methods

### ﻿Morphology analysis

The specimens for this study were collected on 28 September 2024 in Wawu Mountain, Meishan City, Sichuan Province, China. The new species was meticulously dissected in situ. A thorough examination of the morphological characteristics of each part was conducted. It particularly focused on the petiole length of branches and leaves in their natural growth state, the length and width of leaf blades, the number of pairs of leaf veins, the colors of the adaxial and abaxial leaf surfaces, the overall shape of the leaves, the flowers, and the shapes and sizes of the seeds. The holotype voucher specimens were deposited at the botanical herbarium of Sichuan Normal University (SCNU!). The conservation status of the new species was assessed following the guidelines of the IUCN Red List categories and criteria (IUCN 2024). The description and discussion of floral organs and individual development followed the terminology of Yu et al. (2016).

### ﻿DNA extraction and sequencing

Total genomic DNA was obtained using the CTAB method (Doyle 1987). The Illumina DNA Library Construction Guide was followed to construct a paired-end DNA library (Allen et al. 2006). The complete cp genome was sequenced on the Illumina HiSeq XTen platform (San Diego, CA, USA). GetOrganelle v.1.7.7.1 (Jin et al. 2020; Qian et al. 2024) was used to assemble the cp genome, as successfully applied in recent *Impatiens* studies (Wang et al. 2022, Song et al. 2024). The assembly of the genome was verified by Bandage v.0.8.1 (Wick et al. 2015; Zhao et al. 2020). CPGAVAS2 (Shi et al. 2019; Yan et al. 2023) was employed to annotate the genome with manual checking and modification. The final annotated genome was submitted to NCBI GenBank, obtaining the accession number (PQ740019). We further used CPGView (http://47.96.249.172:16085/cpgview/view) to visualize the structure of the cp genome and cis-splicing and trans-splicing (Li et al. 2024).

### ﻿Phylogenetic analysis

**Figure 1.**
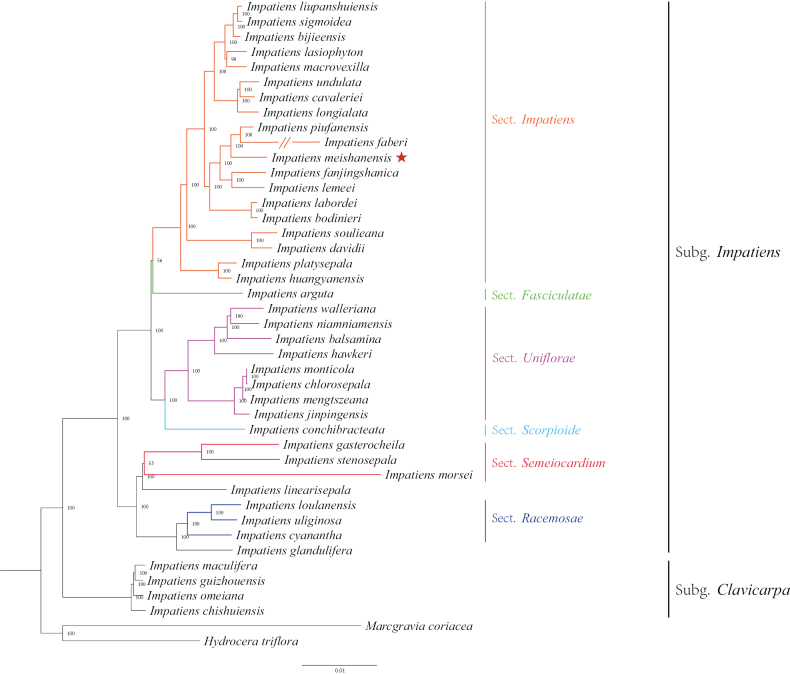
Phylogenetic tree reconstruction of 41 taxa using the ML method, based on complete cp genomes (the phylogenetic position of *Impatiensmeishanensis* is marked with a red star).

A total of 40 cp genomes of *Impatiens* and 2 outgroups (*Hydroceratriflora* (L.) Wight & Arn. and *Marcgraviacoriacea* Vahl) were retrieved and downloaded from the NCBI GenBank database (https://www.ncbi.nlm.nih.gov/Genbank) (Table 1). Multiple sequence comparison was performed with MAFFT v7.526 (Katoh and Standley 2013, Wu et al. 2021). The maximum likelihood (ML) tree was inferred with RAxML-HPC2 on ACCESS v.8.2.12 (Stamatakis 2014), accessible on CIPRES (https://www.phylo.org/) (Miller et al. 2010; Hu et al. 2020), employing the GTRGAMMA model, with bootstrap 1000. Finally, the phylogenetic tree was edited and visualized using FigTree v1.4.4 (Hu et al. 2018) (Fig. 1).

**Table 1.** Accessions sampled for molecular analysis of *Impatiens* and its close relatives.

Species	Family	Genus	GenBank number
*Hydroceratriflora* Wight & Arn.	Balsaminaceae	*Hydrocera*	NC037400.1
*Marcgraviacoriacea* Vahl.	Marcgraviaceae	*Marcgravia*	NC041255.1
*Impatiensbodinieri* Hook.f.	Balsaminaceae	*Impatiens*	PQ452959.1
*Impatienschishuiensis* Y.X.Xiong	Balsaminaceae	*Impatiens*	PP724655.1
*Impatiensfaberi* Hook.f.	Balsaminaceae	*Impatiens*	OR135485.1
*Impatiensbalsamina* L.	Balsaminaceae	*Impatiens*	NC059942.1
*Impatiensmonticola* Hook.f.	Balsaminaceae	*Impatiens*	NC058205.1
*Impatienscyanantha* Hook.f.	Balsaminaceae	*Impatiens*	NC058204.1
*Impatienshawkeri* W.Bull	Balsaminaceae	*Impatiens*	NC048520.1
*Impatiensomeiana* Hook.f.	Balsaminaceae	*Impatiens*	NC072171.1
*Impatienssoulieana* Hook.f.	Balsaminaceae	*Impatiens*	PQ452968.1
*Impatienslongialata* E.Pritz.	Balsaminaceae	*Impatiens*	PQ452967.1
*Impatienssigmoidea* Hook.f.	Balsaminaceae	*Impatiens*	PQ156321.1
*Impatienscavaleriei* X.X.Bai & R.X.Huang	Balsaminaceae	*Impatiens*	PQ156320.1
*Impatienslasiophyton* Hook.f.	Balsaminaceae	*Impatiens*	PQ156319.1
*Impatiensliupanshuiensis* X.X.Bai & T.H.Yuan	Balsaminaceae	*Impatiens*	PQ156318.1
*Impatienslabordei* Hook.f.	Balsaminaceae	*Impatiens*	PQ156317.1
*Impatiensbijieensis* X.X.Bai & L.Y.Ren	Balsaminaceae	*Impatiens*	PQ156316.1
*Impatienshuangyanensis* X.F.Jin & B.Y.Ding	Balsaminaceae	*Impatiens*	OR139616.1
*Impatienswalleriana* Hook.f.	Balsaminaceae	*Impatiens*	NC059949.1
*Impatiensstenosepala* E.Pritz.	Balsaminaceae	*Impatiens*	NC059948.1
*Impatiensloulanensis* Hook.f.	Balsaminaceae	*Impatiens*	NC059947.1
*Impatienslinearisepala* S.Akiyama, H.Ohba & S.K.Wu	Balsaminaceae	*Impatiens*	NC059946.1
*Impatiensguizhouensis* Y.L.Chen	Balsaminaceae	*Impatiens*	NC059945.1
*Impatiensfanjingshanica* Y.L.Chen	Balsaminaceae	*Impatiens*	NC059944.1
*Impatienschlorosepala* Hand.-Mazz.	Balsaminaceae	*Impatiens*	NC059943.1
*Impatiensdavidii* Franch.	Balsaminaceae	*Impatiens*	NC058801.1
*Impatiensglandulifera* Royle	Balsaminaceae	*Impatiens*	NC044718.1
*Impatienspiufanensis* Hook.f.	Balsaminaceae	*Impatiens*	NC037401.1
*Impatiensmorsei* Hook.f.	Balsaminaceae	*Impatiens*	NC071773.1
*Impatiensconchibracteata* Y.L.Chen & Y.Q.Lu	Balsaminaceae	*Impatiens*	NC071771.1
*Impatiensarguta* Hook.f. & Thomson	Balsaminaceae	*Impatiens*	NC071770.1
*Impatiensplatysepala* Y.L.Chen	Balsaminaceae	*Impatiens*	NC068751.1
*Impatiensmacrovexilla* var. yaoshanensis S.X.Yu, Y.L.Chen & H.N.Qin	Balsaminaceae	*Impatiens*	NC060668.1
*Impatiensmengtszeana* Hook.f.	Balsaminaceae	*Impatiens*	NC058215.1
*Impatiensuliginosa* Franch.	Balsaminaceae	*Impatiens*	NC059760.1
*Impatiensgasterocheila* Hook.f.	Balsaminaceae	*Impatiens*	PQ452965.1
*Impatiensjinpingensis* Y.M.Shui & G.F.Li	Balsaminaceae	*Impatiens*	PQ452961.1
*Impatienslemeei* H.Lév.	Balsaminaceae	*Impatiens*	PQ452964.1
*Impatiensmaculifera* S.X.Yu & Chang Y.Xia	Balsaminaceae	*Impatiens*	PQ452960.1
*Impatiensniamniamensis* Gilg	Balsaminaceae	*Impatiens*	PQ452962.1
*Impatiensundulata* Y.L.Chen & Y.Q.Lu	Balsaminaceae	*Impatiens*	PQ452969.1

## ﻿Result and discussion

### ﻿Taxonomic treatment

#### ﻿ Impatiens meishanensis

Taxon classificationPlantaeEricalesBalsaminaceae

K.Huang & Z.X.Fu sp. nov.

0BBCEA65-16C4-5142-B9B9-D638DDE02CD8

urn:lsid:ipni.org:names:77366288-1

##### Type.

**Figure 2.**
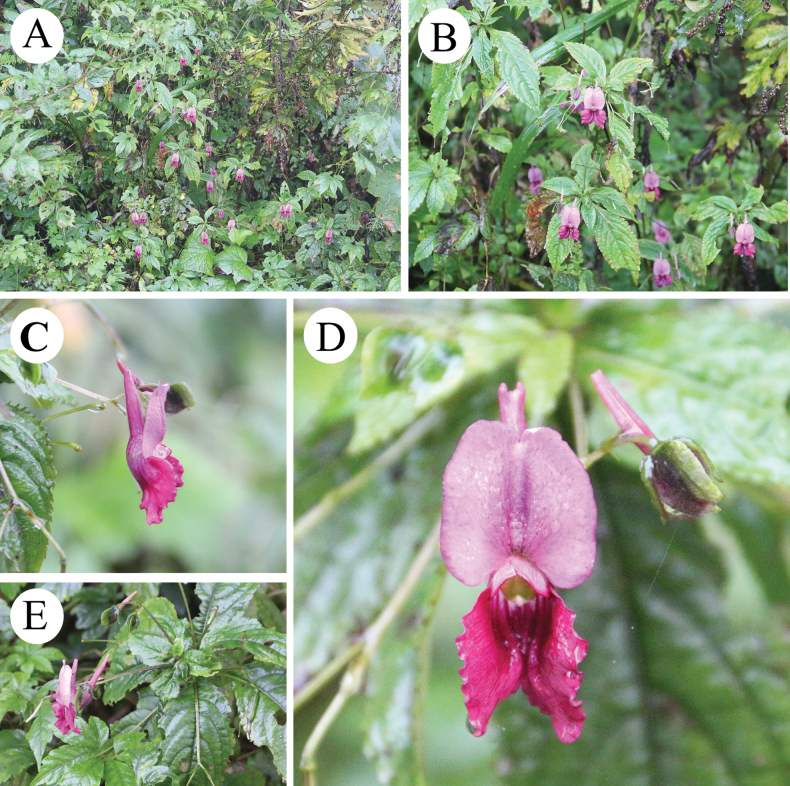
*Impatiensmeishanensis* K.Huang & Z.X.Fu, sp. nov. **A.** Habit; **B.** Population; **C.** Flower in lateral view; **D.** Flower in face view; **E.** Plant. Photographs by Dr. Zhixi Fu.

**Figure 3.**
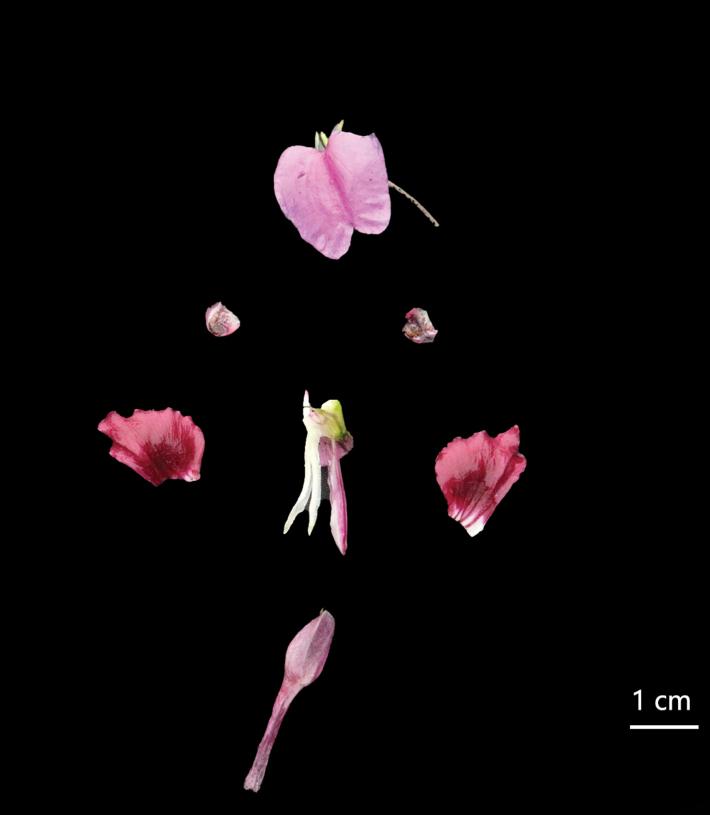
*Impatiensmeishanensis* K.Huang & Z.X.Fu, sp. nov. Flower dissected. Photographed by Dr. Zhixi Fu.

**Figure 4.**
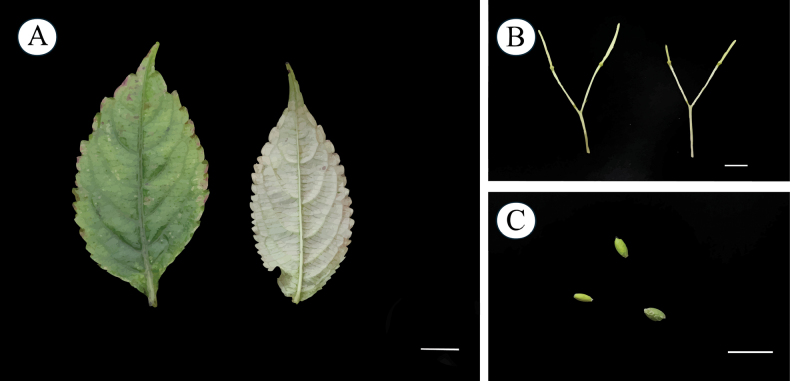
*Impatiensmeishanensis* K.Huang & Z.X.Fu, sp. nov. **A.** Leaves; **B.** Capsules; **C.** Seeds. Photographed by Dr. Zhixi Fu. Scale bars: 1 cm.

**Figure 5.**
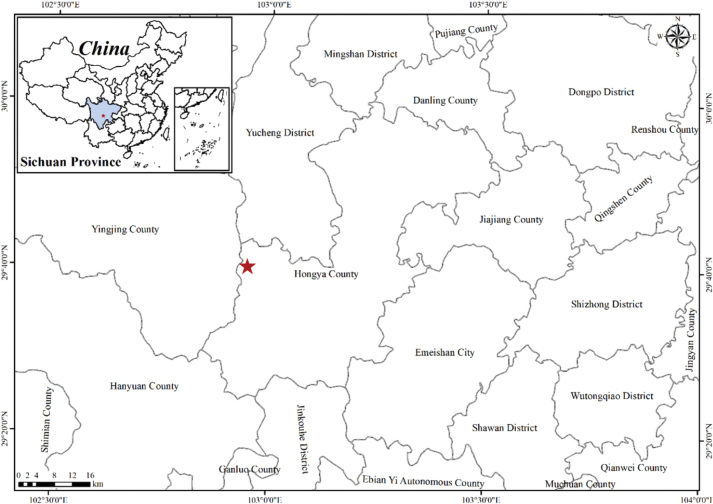
Holotype image of *Impatiensmeishanensis* K.Huang & Z.X.Fu, sp. nov.

China: • Sichuan Province, Meishan City, Wawu Mountain, growing on rocky soil by a mountain road, 1991.5 m.a.s.l., 29.6702°N, 102.9492°E, 28^th^ September 2024 (fl.), *Yanru Zhang & Zhixi Fu 688* (holotype: SCNU! [SCNU00008]; isotype: [SCNU00009]) (Figs 2–5).

##### Diagnosis.

This new species is most closely related to *I.faberi*, followed by *I.piufanensis*, sharing a common ancestral node, with alternate, ovate leaves, apex acuminate, base cuneate, and margin serrate. However, it differs in being almost glabrous throughout, with the spur nearly parallel to the dorsal petal, and the seeds ellipsoid, coats with reticulate ornamentation (Figs 2–5).

##### Description.

Herbs perennial, 40–80 cm tall, glabrous throughout. ***Stem*** erect, branched at the base. ***Leaves*** alternate, petiolate, petiole length 0.5–2 cm. Leaf blade ovate, 5–12 × 2–3 cm in size, apex acuminate, base cuneate or rounded, margin serrate or undulate, dark green adaxially and grey-green abaxially, glabrous on both surfaces, with pinnate reticulate veins and conspicuous lateral veins in about 5–7 pairs. ***Peduncle*** arising from upper leaf axils, 5–10 cm, fine, erect, glabrous or puberulent, 2-flowered, bracts above basal pedicels. ***Bracts*** lanceolate, glabrous. ***Flowers*** large, ca. 4 cm long, pinkish-purple, with distinctive bright colors, surface smooth. ***Inflorescences*** racemose, 2-flowered. ***Dorsal petal*** 1, suborbicular, ca. 2 × 1.5 cm, apex obtuse, upper and lower bases depressed, with obvious midrib, broadly winged. ***Lower petals*** 2, sessile, 8–13 × 6–10 mm, margins wavy, abaxially slightly thickened at midrib, base whitish, with purple veinlets. ***Lateral sepals*** 2, ovate-oblong, conchate, ca. 4–5 mm long, 2–3 mm wide, apex mucronate. ***Lower sepal*** gable-boat-shaped, ca. 1.5–3 cm long, ca. 1.5 cm deep, mouth obliquely upward, extending backward into a spur, apices blunt and straight. ***Anthers*** acuminate. ***Ovary*** fusiform, erect. ***Capsule*** linear, 3–5 cm long. ***Seeds*** ellipsoid, coats with reticulate ornamentation (Figs 3, 4).

##### Phenology.

This new species of *Impatiens* flowers from August to October each year.

##### Distribution and ecology.

**Figure 6.**
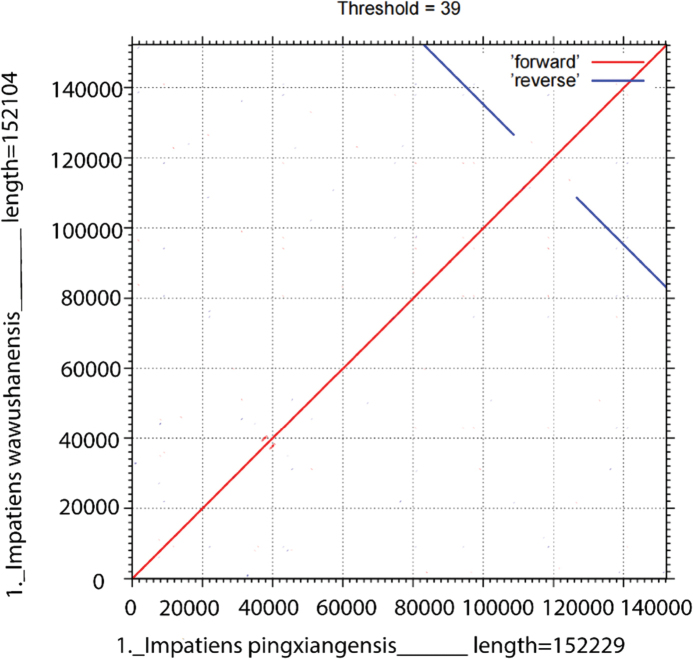
Location of the population of *Impatiensmeishanensis* at Wawu Mountain, Meishan City, and Sichuan Province (red star).

This new species of *Impatiens* grows alongside an undeveloped trail at the lower station of the Gufoping cableway in Wawu Mountain, Sichuan Province (Fig. 6). The new species may grow elsewhere as well, with a high probability of growing on moist, rocky soils in forests.

##### Etymology.

The epithet “meishanensis” is derived from Meishan City, located in Sichuan Province, China.

##### Conservation status.

*I.meishanensis* is a species of *Impatiens* from Meishan City, Sichuan Province, which is currently distributed only in the scenic area of Wawu Mountain and in small numbers. Since Wawu Mountain is a famous scenic area in Sichuan Province with high anthropogenic disturbance, it is recommended that the conservation status of this species be assessed as Data Deficient (DD) according to the IUCN Red List criteria (IUCN 2024).

##### Relationship with related species.

Critical examination of collected specimens, comparison with type material of allied taxa, and relevant taxonomic literature revealed that *I.meishanensis* belongs to a new member of the sect. Impatiens of the subgen. Impatiens. *I.meishanensis* is the closest relative to *Impatiensfaberi*. Morphologically, *I.meishanensis* shares certain similarities with *I.faberi*. However, *I.meishanensis* differs from *I.faberi* in featuring the lower sepal as gable-boat-shaped (vs. funnel-shaped), spur straight (vs. curved), flower large, ca. 4 cm long (vs. ca. 3 cm long), leaves ovate, 5–12 × 2–3 cm (vs. ovate-lanceolate or lanceolate, 5–15 × 2.5–4.5 cm), dorsal petal suborbicular (vs. orbicular), capsule linear (vs. narrowly linear), and seeds ellipsoid, coats with reticulate ornamentation (vs. oblong, smooth). This new species of *Impatiens* is similar to *I.distracta*, *I.faberi*, and *I.piufanensis* (Chen et al. 2007). *I.meishanensis* differs from *I.distracta* in featuring a plant height of 40–80 cm (vs. 30–60 cm), flower large, ca. 4 cm long (vs. small, ca. 3 cm long), leaves ovate, 5–12 × 2–3 cm (vs. ovate-oblong, 5–10 cm), dorsal petal suborbicular (vs. orbicular), bracts glabrous (vs. puberulent), peduncle 4–8 cm (vs. 2–3 cm) and seeds ellipsoid, coats with reticulate ornamentation (vs. oblong, glabrous). To better distinguish the new species morphologically, we list more details in Table 2.

**Table 2.** Comparison of morphological characters among *Impatiensmeishanensis*, *I.faberi*, *I.distracta*, and *I.piufanensis*.

Characters	*I.meishanensis*	*I.faberi*	*I.distracta*	*I.piufanensis*
Plant height	40–80 cm	60–70 cm	30–60 cm	20–40
Shape of leaves	ovate	ovate-lanceolate or lanceolate	ovate-oblong	Long, ovate, or lanceolate
Leave size	5–12 x 2–3 cm	5–15 x 2.5–4.5 cm	5–10 cm	3–6 x 1.2–2.5 cm
Leave margin	serrated or undulating	serrated or crenellated serrations	crenate teeth or crenate serrations	serrate
Lateral veins	5–7 pairs	5–8 pairs	6–7 pairs	4–5 pairs
Flower size	large, 4 cm long	large, 3 cm long	Small, 2 cm long	large, 3 cm long
Dorsal petal	suborbicular, 12–18 mm, concave or 2-cleft at the top, blunt, deeply bifid at the base, with thickened mid-rib on the back, with wings	orbicular, 13–17 mm, concave or 2-cleft at the top, blunt, deeply bifid at the base, with thickened mid-rib on the back, with wings	orbicular, 14 mm, base concave, tip rounded, with thickened mid-rib on the back, broadly winged, wings apically beaked	orbicular or obovate, back middle rib keeled, apex mucronulate
Lower sepal	gable-boated, 1.5–3 cm long, mouth obliquely upward, extending backward into a spur, apices blunt and straight.	angular, 3–4 cm, mouth is oblique, with a small point, bending inwards or straight from the middle	gable boat-shaped, ascending, 1.5 cm long, mouth obliquely upward, apex acute, base narrowed into a thick, obtuse, slightly recurved spur shorter than the gable	funnel-shaped, base decurrent into a curved spur of finely spaced
Spur	straight	curved	slightly curved	curved
Capsule	linear	narrowly linear	linear	linear
Seed	ellipsoid, 3–4 mm long, coats with reticulate ornamentation	oblong, 4 mm long, smooth	ovate-oblong, 3.5–4 mm long, glabrous	subglobular, 3 mm in diameter, smooth
Inflorescences	2-flowered	2-flowered	2-flowered	1-flowered
Lateral sepals	ovate-oblong, conchate, 4–5 x 2–3 mm, apex mucronate	green, ovate, 6–8 x 3–5 mm, with 3–5 veins and a thickened mid-rib	ovate, 7 mm long, apically acuminate, apiculate, 3-veined, glabrous or pubescent	ellipsoid, 5 mm long, apex beaked
Bracts	glabrous	glabrous	puberulent	/
Peduncle	4–8 cm	5–10 cm	2–3 cm	4–5 cm

### ﻿Molecular phylogeny

**Figure 7.**
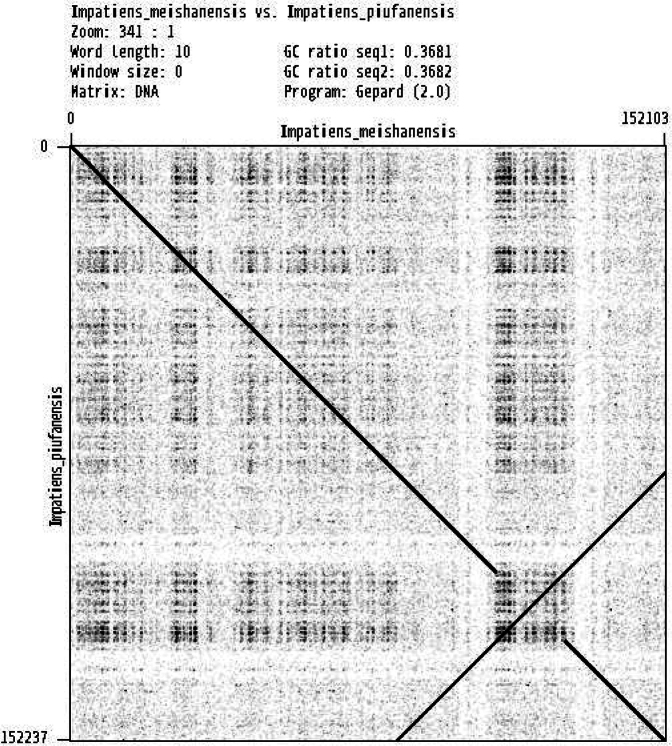
The covariance analyses of two species.

**Figure 8.**
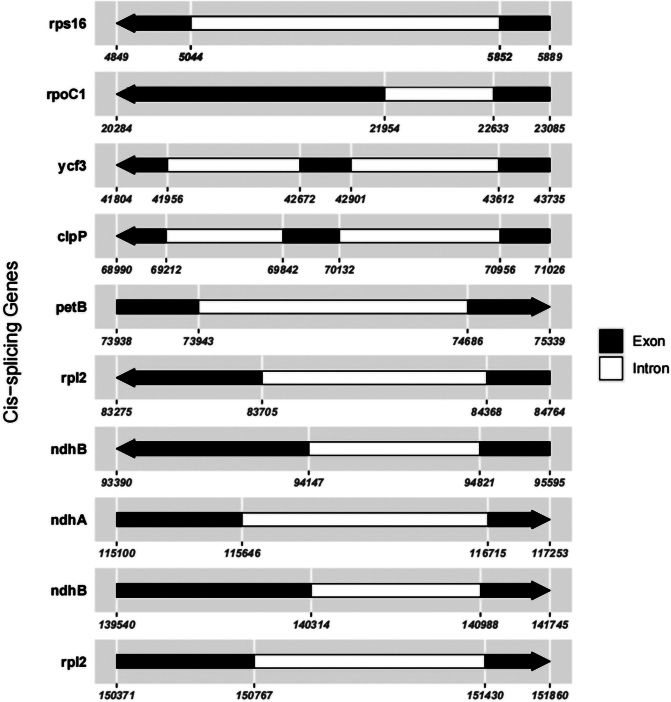
Circular map of *Impatiensmeishanensis*. The map of the complete cp genome was generated using CPGView.

**Figure 9.**
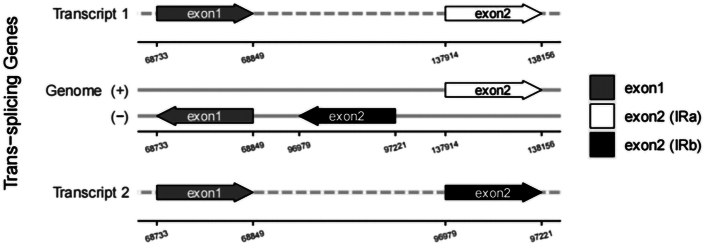
Schematic map of the cis-splicing genes in the cp genome of *Impatiensmeishanensis*.

**Figure 10.**
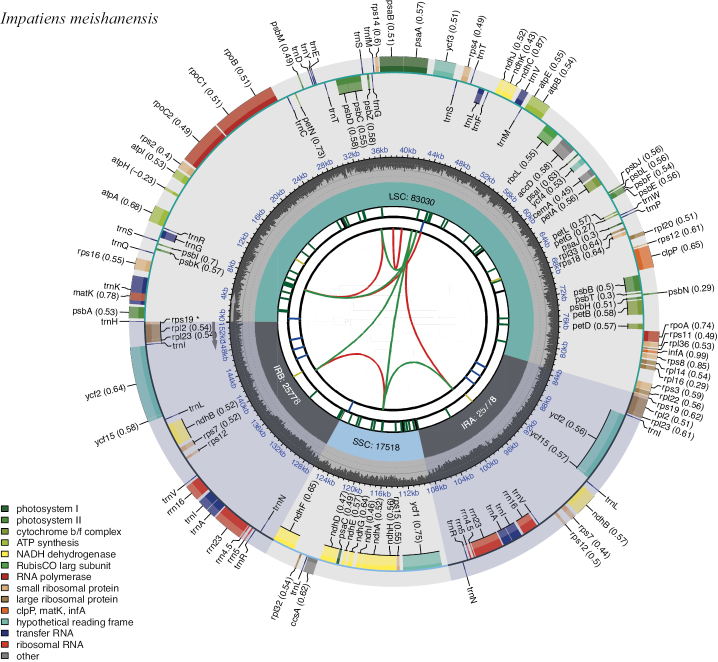
Schematic map of the trans-splicing genes in the cp genome of *Impatiensmeishanensis*.

**Figure 11.**
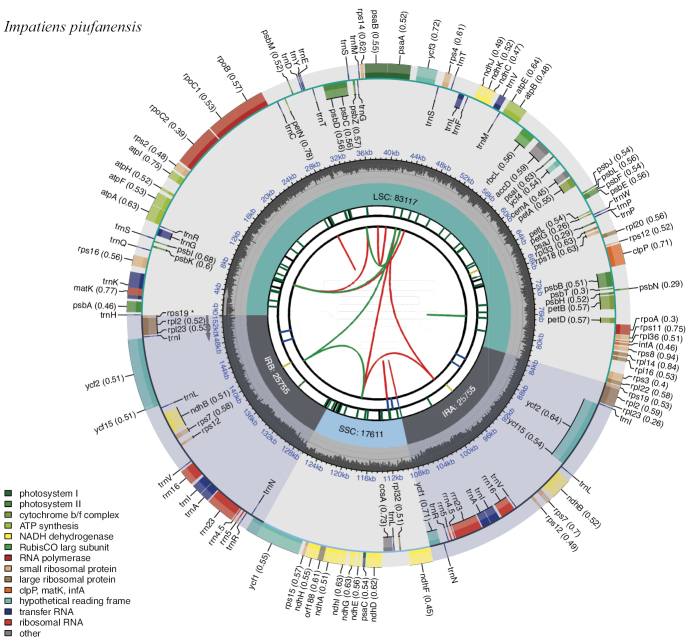
Circular map of *Impatienspiufanensis* (GenBank accession numbers: NC037401.1. The map of the complete cp genome was generated using CPGView.

**Figure 12.**
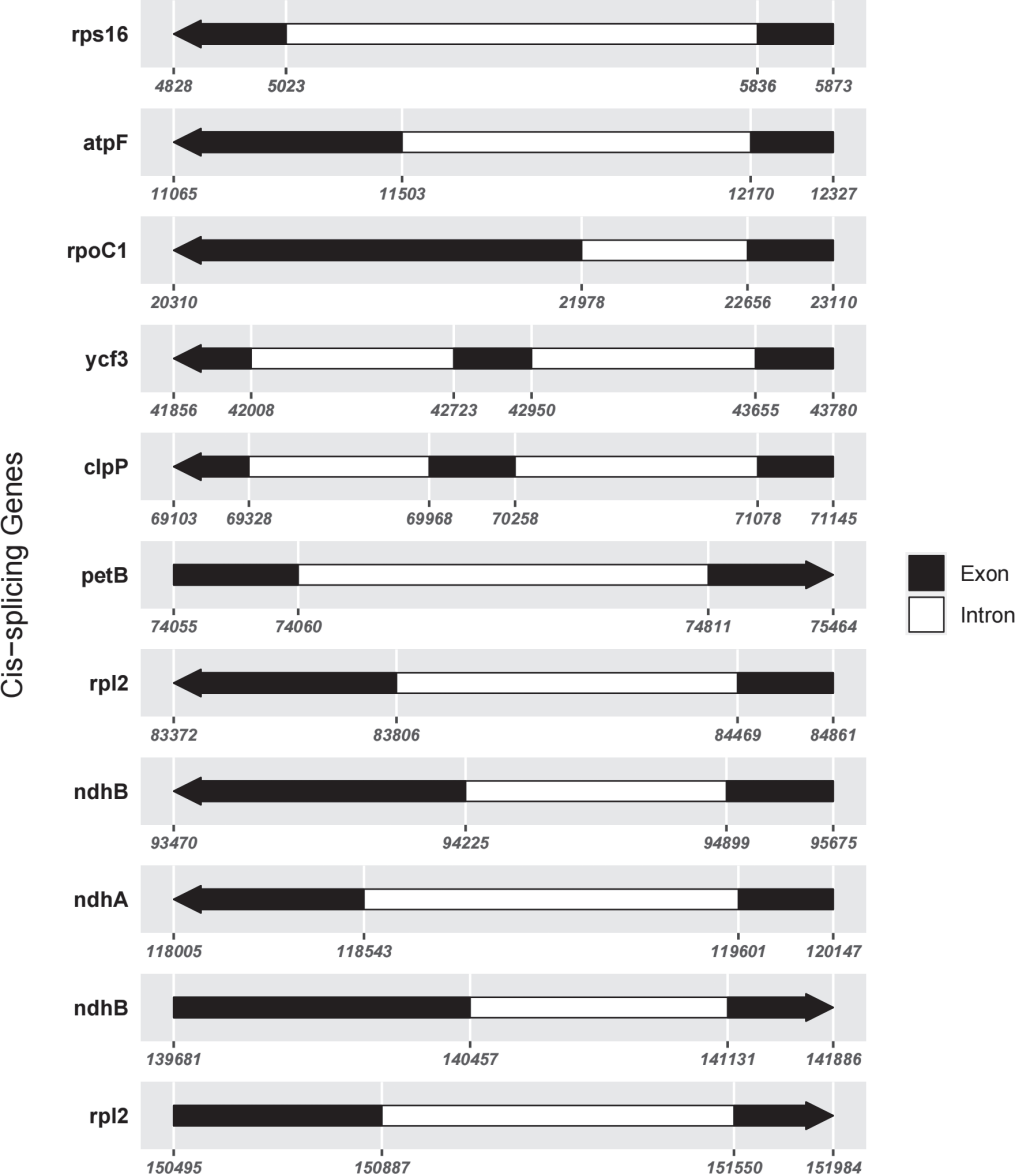
Schematic map of the cis-splicing genes in the cp genome of *Impatienspiufanensis* (GenBank accession numbers: NC037401.1).

**Figure 13.**
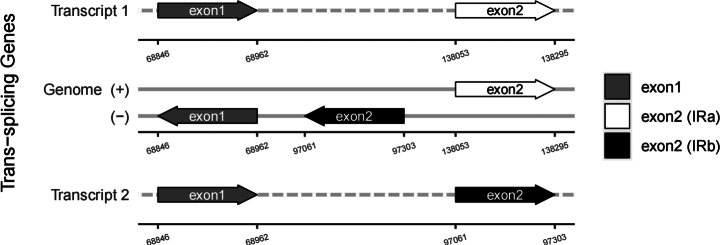
Schematic map of the trans-splicing genes in the cp genome of *Impatienspiufanensis* (GenBank accession numbers: NC037401.1).

In this study, an ML phylogenetic tree was constructed using the cp genome sequences of 40 species of *Impatiens*, two outgroups, and new species (Table 1) (Fig. 1), which were classified according to Yu et al. (2016). This new species is most closely related to *I.faberi*, followed by *I.piufanensis*, but also on different branches. Since the molecular data of *I.faberi* is not validated on NCBI, this study was conducted with *I.piufanensis* for cp genome comparison. The character of genomes is not similar in the two species (Table 3). The complete cp genome of *I.meishanensis* is 152,104 bp, with the GC content of 36.81% (Fig. 8). The LSC length of *I.meishanensis* is 83,030 bp, the SSC length is 17,518 bp, and the IR length is 51,556 bp. In the cp nucleotide sequence of *I.meishanensis*, the base numbers of A, T, C, and G are 47,508, 48,610, 28,254, and 27,732, respectively. The complete cp genome of *I.piufanensis* is 152,238 bp, with the GC content of 36.82% (Fig. 11). The LSC length of *I.piufanensis* is 83,117 bp, the SSC length is 17,611 bp, and the IR length is 51,510 bp. In the cp nucleotide sequence of *I.piufanensis*, the numbers of A, T, C, and G are 47,508, 48,672, 28,520, and 27,536, respectively. The differences in LSC/SSC/IR lengths and nucleotide composition may reflect evolutionary adjustments. These complete cp genome sequences are aligned by Gepard v.2.1.0 (Krumsiek et al. 2007) (Fig. 7). The Gepard dot plot (Fig. 7) confirms high synteny between *I.meishanensis* and *I.piufanensis*cp genomes, supporting their close relationship. The cis-splicing genes are the same, but their positions in the sequence are different (Figs 9, 12). The rps12 is a trans-splicing gene (Figs 10, 13).

**Table 3.** Comparative analyses of cp genomes between *Impatiensmeishanensis* and *I.piufanensis*.

Species	Genome Size (bp)	LSC (bp)	IR (bp)	SSC (bp)	A	T	C	G	GC Content (%)
*I.meishanensis*	152,104	83,030	51,556	17,518	47,508	48,610	28,254	27,732	36.81
*I.piufanensis*	152,238	83,117	51,510	17,611	47,508	48,672	28,520	27,536	36.82

The ML phylogenetic tree (Fig. 1) strongly supports the distinct taxonomic status of *I.meishanensis* as a new species, clustering closely with *I.faberi* and *I.piufanensis* but forming an independent branch. Despite the close genetic affinity, several lines of evidence highlight their divergence. While *I.meishanensis* shares certain floral traits with *I.piufanensis*, it differs in key characteristics such as leaf shape, lower sepal, and seed structure (Table 2). These morphological disparities align with the observed molecular divergence, suggesting ecological adaptation or reproductive isolation.

## Supplementary Material

XML Treatment for Impatiens meishanensis

## ﻿Additional information

### Use of AI

No use of AI was reported.

### Author ORCIDs

Yanru Zhang https://orcid.org/0009-0009-0450-2001

Xinyu Chen https://orcid.org/0009-0008-6347-2490

Tianmeng Qu https://orcid.org/0009-0008-0185-5306

Xinyi Zheng https://orcid.org/0009-0003-2300-5525

Bo Li https://orcid.org/0000-0002-5131-8639

Jieli Yue https://orcid.org/0009-0002-8786-2473

Xiaoqin Liu https://orcid.org/0009-0007-8195-0444

Min Pan https://orcid.org/0009-0008-2123-4232

Ke Huang https://orcid.org/0009-0006-2057-5452

Zhixi Fu https://orcid.org/0000-0002-2789-6287

### Data availability

All of the data that support the findings of this study are available in the main text.
